# Malnutrition in pregnancy following bariatric surgery: three clinical cases of fetal neural defects

**DOI:** 10.1186/1475-2891-13-59

**Published:** 2014-06-14

**Authors:** Gloria Pelizzo, Valeria Calcaterra, Mario Fusillo, Ghassan Nakib, Antonio Maria Ierullo, Alessandro Alfei, Arsenio Spinillo, Mauro Stronati, Hellas Cena

**Affiliations:** 1 Department of Maternal and Children’s Health, Pediatric Surgery Unit, Fondazione IRCCS Policlinico San Matteo and University of Pavia, P.le Golgi 2, Pavia 27100, Italy; 2 Department of Internal Medicine, University of Pavia and Department of Maternal and Children’s Health, Pediatric Unit, Fondazione IRCCS Policlinico San Matteo Pavia, P.le Golgi 2, Pavia 27100, Italy; 3 Department of Maternal and Children’s Health, Obstetrics and Gynecology Unit, Fondazione IRCCS Policlinico San Matteo Pavia and University of Pavia, P.le Golgi 2, Pavia 27100, Italy; 4 Department of Maternal and Children’s Health, Neonatal Intensive Care Unit, Fondazione IRCCS Policlinico San Matteo Pavia, P.le Golgi 2, Pavia 27100, Italy; 5 Department of Public Health, Neurosciences, Experimental and Forensic Medicine, Section of Human Nutrition, University of Pavia, Cascina Cravino, Pavia 27100, Italy

**Keywords:** Maternal malnutrition, Obesity, Bariatric surgery, Neural, Malformations

## Abstract

**Objective:**

Bariatric surgery results in decreased food intake and a variable degree of malabsorption. Without adequate supplementation, the most common complications of this surgery are nutritional disorders. Pregnancy following surgery for obesity is a particular condition requiring strict monitoring of nutrient intake necessary for fetal development and a favourable neonatal prognosis.

**Patients:**

Malnutrition in pregnancy and congenital neural malformations are reported in three women who had previously undergone bariatric surgery (1, 5 and 18 years before pregnancy, respectively). Two patients underwent the Roux en Y bypass and one bilio-pancreatic diversion with gastroplasty. None of the three received pre-conceptional nutritional counselling. Patients 1 and 2 did not undergo postoperative nutritional surveillance; nutrient supplementation was started at 22 and 20 weeks gestation, respectively. In patient 3, supplementation was stopped at six weeks gestation.

**Results:**

Newborns 1 and 2 presented with dorsal myelomeningocele and ventricular dilation. Both underwent surgery and a ventriculo-peritoneal shunt was inserted in the first month of life. Newborn 3 had microcephaly, bilateral microphthalmia and sensorineural deafness.

**Conclusions:**

Diet and nutritional status, before and during pregnancy, play an important role in the early processes of fetal development and neonatal outcome. Women of childbearing age who have had bariatric surgery, should be encouraged to follow a well-balanced diet as part of a weight management strategy. They should be advised to take recommended maternal supplements.

## Background

Obesity has reached epidemic proportions worldwide [1]. About 25% of women meet obesity criteria and one third are of reproductive age [2]. Obesity in pregnancy represents a serious problem, because of the adverse effects on maternal and neonatal outcomes [3]. Bariatric surgery represents an alternative option for morbid obesity in those patients who have not achieved adequate weight loss with lifestyle and medical management, and/or those who suffer from complications of obesity. Surgery to treat obesity should really be reserved for those who will adhere to the lifestyle changes that are necessary for a successful outcome All patients should meet the appropriateness criteria for bariatric surgery [3-8].

There are three main types of bariatric surgical procedures: restrictive, combined (restrictive and malabsorptive) and primarily malabsorptive. The Roux-en-Y gastric bypass and biliopancreatic diversion more effectively maintain durable weight loss.

Pregnancies following bariatric surgery are generally considered safe. Weight reduction after surgical treatment results in a significantly lower incidence of severe obesity-related complications such as hypertension, gestational diabetes, large-for-gestational age infants and caesarean section. Nevertheless, increased incidences of miscarriage, prematurity and growth restriction have been reported in these patients [3,4].

Malabsorption following bariatric surgical procedures and malnutrition may contribute to the risk for birth defects [3,9-11], such as neural tube defects [12,13]. Multiple genetic and environmental factors, including maternal nutrition, regulate survival and development of the embryo in early gestation [14-16].

Maternal malnutrition in pregnancy and fetal neural deformities are reported in three patients who previously underwent bariatric surgery. The importance of an early clinical nutritional evaluation by a multidisciplinary team in women undergoing surgery for obesity is highlighted.

### Case presentations

#### Case 1

A 40 year old female became pregnant one year following a Roux-en-Y gastric bypass (Table 1). Postoperative nutritional surveillance, pre-conceptional nutritional counselling and nutrient supplementation were not adopted.

**Table 1:** Clinical and anthropometric data of the three mothers

	**MOTHER 1**	**MOTHER 2**	**MOTHER 3**
Age at bariatric surgery (years)	39	22	17
Co-morbidities at surgery	no	no	no
Bariatric procedure	roux en Y gastric bypass	roux en Y gastric bypass	bilio-pancreatic diversion with gastroplasty
BMI at bariatric surgery (kg/m^2^)	40	40.5	48
Pre-gravidic BMI (kg/m^2^)	23.4	25.4	24.2
Age at pregnancy (years)	40	27	35
Time interval between bariatric surgery and pregnancy (years)	1	5	18

She was referred to our Department during the second trimester of gestation after the detection of fetal neural tube defect (Table 2). Clinical and nutritional assessments were made by collecting anthropometric data, biochemical laboratory data, diet and medical history, and physical evaluation (Table 3). Nutritional supplementation with vitamins and minerals was started at 22 weeks’ gestation.

**Table 2:** Fetal and neonatal outcome

	**CASE 1**	**CASE 2**	**CASE 3**
**FETAL OUTCOME**			
**Gestational age at diagnosis (weeks + days)**	22 + 0	20 + 5	20 + 3
**Ultrasound findings**			
**Indirect signs**			
Deformation of frontal cranial bones (lemon sign)	Present	Present	Absent
Abnormal curvature of cerebellum (banana sign)	Present	Present	Absent
Ventricle dilatation (site; grade)	Bilateral; Moderate	Bilateral; Moderate	Absent
Hypoplastic posterior cranial fossa	Present	Present	Absent
**Direct signs**			
Spinal deformation (level of lesion)	Lumbar L1-L5	Thoracic Lumbar (T9-S1)	Absent
Myelomeningocele (dimension mm)	Present (27x28)	Present (54x37)	Absent
**Other associated malformations**	Sacral Agenesia	Hemivertebrae T5	Absent
**Magnetic resonance imaging**			
Findings	Moderate bilateral ventriculomegaly (Arnold Chiari Type II); spina bifida extending from T1-T5	Moderate bilateral ventriculomegaly (Arnold Chiari Type I); spina bifida extending from T9-S1	Not Performed
**NEONATAL OUTCOME**			
**Sex**	F	F	M
**Gestational age at birth (weeks; days)**	38	38	34 + 6
**Mode of delivery**	Elective Caesarean Section	Elective Caesarean Section	Vaginal
**Apgar score**			
1 minute	6	8	5
5 minutes	7	9	7
**Weight at birth (gr)**	2700	3090	2287
**Surgery**	Closure MMC + DVP	Closure MMC + DVP	-

MMC = myelomeningocele; DVP = drainage ventriculo-peritoneal.

**Table 3:** Maternal nutritional findings during pregnancy

	**MOTHER 1**	**MOTHER 2**	**MOTHER 3**
Clinical signs	fatigue	fatigue	nyctalopia
Nutritional supplements before pregnancy	no	no	yes
Nutritional supplements during first trimester	no	no	no
Nutritional status during pregnancy (gestational age)	24 w	20 w	22 w
-Folates (ng/ml; nv 2–19.9)	5.1	>24	*
-Vitamin B12 (pg/ml; nv 243–894)	201	<15	*
-Vitamin A (mcg/ml; nv 0.25-0.86)	0.24	0.1	*
-1,25-dihydroxyVitD (pmol/L; nv 48–110)	44.6	40.3	*
-25-hydroxyVitD (nmol/L; nv 23–113)	40.30	76.28	*
-Iron (mcg/dl; nv 25–156)	16	73	*
-Ferritin (ng/ml; nv 18–440)	2	10	*
-Hb (g/dl; nv 11.7-15.5)	8.7	12	*
-Pre-albumin levels (mg/dl; nv 20–40)	19	17	-

nv = normal value range.

*hypovitaminosis as reported by the patient. Clinical records not available.

The newborn underwent surgery on the first day of life for posterior myelomeningocele after MRI evaluation. A ventriculo-peritoneal shunt was positioned in the first month of life to treat ventricular dilation (Table 2).

#### Case 2

A 22 year old female underwent an uncomplicated laparoscopic Roux-en-Y gastric bypass for obesity five years prior to becoming pregnant (Table 1). This patient was not evaluated by a nutritionist either in the postoperative period or during the pre-conceptional phase. Nutritional supplementation was not adopted.

She was admitted to our Institution during the second trimester of pregnancy after detecting a thoraco-lumbar fetal neural tube defect on ultrasound (US) (Table 2). Anthropometric and biochemical laboratory data, diet and medical history, physical evaluation were collected to perform the clinical and nutritional assessment (Table 3). Nutrient supplementation was immediately introduced during the 20^th^ week.

At birth, prenatal findings of myelomeningocele extending from T9-S1 were confirmed by MRI (Table 2). Closure of myelomeningocele was performed at the first day of life and a ventriculo-peritoneal drainage tube was positioned one month later.

#### Case 3

A 35 year old woman became pregnant 18 years after bariatric surgery consisting of bilio-pancreatic diversion with gastroplasty (Table 1). She was referred at the third trimester of pregnancy for fetal microcephaly. The patient reported stopping nutritional supplementation at six weeks gestation. Moreover, she refused nutritional care and supplements prescribed after bariatric surgery (BS) until she had two spontaneous abortions 16 years post BS. Nutritional data of the patient are reported in Table 3.

The newborn male at 35 weeks gestation weighing 2287 g, presented with microcephaly, bilateral micro/anophthalmia, skeletal dysplasia, short limbs and persistent ductus arteriosus. Bilateral microphthalmia with optic nerve, chiasm and tract hypotrophy were documented by MRI. Sensorineural deafness was also diagnosed. Infectious and genetic causes of microphthalmia were excluded. He underwent positioning of a prosthetic eye and cochlear devices at 5 and 16 months, respectively. A gastrostomy feeding tube was also inserted during the first year of life to treat his failure to thrive; enteral nutrient supplementation for the infant was stopped at two years of age.

Clinical, nutritional and anthropometric data of the mothers are reported in Tables 1 and 3.

In Table 2, prenatal imaging and neonatal outcome are provided.

## Conclusions

Maternal nutritional status may be involved in the etiology of fetal neural tube defects (NTDs). In this report, we describe micronutrient and/or vitamin deficiency in three pregnant women who had previously undergone bariatric surgery and gave birth to progeny with congenital neural defects.

Any bariatric procedure may lead to an increased risk of nutritional deficiency during the postoperative period [17]. The mechanism of malnutrition following bariatric procedures is quite complex. Risk factors may include preoperative malnutrition (e.g., vitamin D, iron), decreased food intake (dumping syndrome, reduced hunger and increased satiety, food intolerances), inadequate nutrient supplementation (poor compliance with multivitamin/multimineral regimen, insufficient amounts of vitamins and/or minerals in supplements) and/or nutritional support (lack of follow-up, insufficient monitoring, difficulty in recognizing symptoms of deficiency), nutrient malabsorption (reduced absorptive gastro-intestinal area) [18,19].

All patients after bariatric surgery should take vitamins and microelements to prevent micronutrient deficiencies. Unfortunately, more than 40% of women after these surgical procedures do not take multiple vitamin supplements for long periods of time [2], with a consequent high risk for malnutritional complications, especially during pregnancy when an increased requirement for microelements and vitamins is recognized.

Women should be instructed to avoid pregnancy for 12 – 24 months following bariatric surgery, since this is the period of most rapid weight loss [8,11]. If an unplanned pregnancy occurs during this period, nutritional assessment should be carried out immediately and frequently monitored during the entire gestational period, especially when deficiencies are detected. Nutrients such as vitamins A, B6, B12 and folic acid and zinc affect embryogenesis and a deficiency in any of these elements may be related to spontaneous abortion failure and to a spectrum of neurodevelopmental disorders [16].

Brain and spinal cord development arises early in pregnancy (18–28 days) from specialised cells. The timing of their development enhances the importance of adequate maternal nutrition before gestation and in the first trimester [20-22]. In particular, folic acid, vitamin B12 and vitamin A and its biologically active metabolite retinoic acid are thought to be involved in neurulation and subsequent neural tube growth and patterning [20].

Fetal neurologic malformations result from failed neural tube closure and/or neural crest migration.

Neural tube defects (NTDs) are common congenital malformations of the central nervous system. NTDs resulting from failed complete neural tube closure at the rostral or caudal end lead to anencephaly and spina bifida [21]. In cases 1 and 2, dorsal myelomeningocele resulted from a defect in primary neurulation including exposition of neural tissue and cerebrospinal fluid leakage. The total prevalence of NTD-affected pregnancies ranges between 0.4 and 2 per 1000 in European countries. The NTD process involves multiple genes, nutritional and environmental factors [23], and seems to be related to defects in the folate-methionine metabolic pathway, since vitamin B12 and folic acid preconception supplementation has been proven to substantially reduce the risk of having an NTD affected pregnancy [15,22-24].

Neural crest cell migration defects contribute to malformation of the optic and acoustic pathways. Signs of congenital ocular abnormalities such as microphthalmia, characterized by abnormally small eyes with or without structural abnormalities, may depend on vitamin A deficiency leading to a disruption of ocular development [20,25]. Anophthalmia/microphthalmia have their genesis in early gestation and are the result of failure of the anterior neural tube or optic pits to enlarge and form optic vesicles or degeneration and disappearance of the optic vesicle [26]. When optic nerve, chiasm and/or tracts with micro/anophthalmia are observed this may indicate the regression of a partially developed eye rather than aplasia of the optic vesicle. The prevalence of anophthalmia and microphthalmia has been estimated to be 3 and 14 per 100,000 births, respectively [27].

The precise pathogenesis of these conditions remains unknown, but non-genetic and non-infectious causes have been postulated and include maternal vitamin A deficiency [25]. It has been demonstrated that in animal models, deprivation of retinoic acid (RA) during eye development causes microphthalmia as well as other visual defects [20] and the timing of the RA deficiency is critical to developmental outcome [28]. Glichrist H et al. [29] and Smets et al. [30] described two cases of microphthalmia and ocular malformations in infants whose mothers had undergone biliopancreatic diversion surgery for obesity with documented hypovitaminosis A.

The clinical evidence of nyctalopia in our third case is thought to be related to hypovitaminosis A, as reported by the patient. The baby also exhibited complex congenital malformations including microphthalmia and optic nerve, chiasm and tract hypoplasia; this condition might be correlated to abnormal neural crest cell migration or to degeneration of the optic vesicle induced by vitamin deficiency [25].

A multidisciplinary health care approach in this setting should include surgeons, nutritionists, obstetricians, endocrinologists, cardiologists and primary care physicians. Nutritional counseling, for assessment and supplementation, should be performed before and following bariatric surgery to reduce the related risks of fetal malformations.

Protein, iron, folate, calcium, vitamin B_12_ and D are the most common nutrient deficiencies following procedures that induce malabsorption [17]. Pre and postconception micro-nutrient status assessment is strongly recommended in women who have undergone bariatric surgery. Nutritional therapy should be initiated right away upon any nutrient deficit detection. When no deficits are detected, a complete blood count and measurement of iron, ferritin, folic acid, vitamin B12, vitamin A, 25-hydroxyvitamin D, calcium, and parathyroid hormone levels should be performed every trimester.

Nutrient deficiencies may also occur after restrictive surgical procedures, such as adjustable gastric banding, because of decreased food intake and dietary imbalances. There is no consensus on the management of pregnant women who undergo this procedure, but early consultation with a clinical nutritionist is recommended.

Lifelong nutritional education and lifestyle changes in women of childbearing age following bariatric surgery are essential for a successful pregnancy outcome. The main goals of medical nutrition therapy during pregnancy are to ensure acceptable weight gain, promote fetal growth and development and provide adequate vitamin, mineral and protein intake. Former recipients of bariatric surgery are recommended to continue supplements before and during pregnancy. Educational support and assessments to verify nutritional requirements are also strongly recommended.

### Consent

Written informed consent was obtained from the patients for the publication of this case report.

## Abbreviations

BS: Bariatric surgery; NTDs: Neural tube defects; RA: Retinoid acid.

## Competing interests

The authors declare that they have no competing interests.

## Authors’ contributions

GP: provided surgical support and contributed to the writing of the final manuscript. VC: provided pediatric support and contributed to the writing of the manuscript draft. MF: provided surgical support. GN: provided surgical support and contributed to the writing of the manuscript draft. AMI: provided gynecological support and contributed to the writing of the manuscript draft. AA and AS: provided gynecological support. MS: provided neonatal support. HC: provided nutritional support and contributed to the writing of the manuscript draft. All authors read and approved the final manuscript.
